# Linking categorical models for prediction of pleasantness score using individual predictions of sweetness and creaminess: An advancement of categorical modeling

**DOI:** 10.1007/s10928-021-09771-y

**Published:** 2021-07-01

**Authors:** Jennifer Leohr, Maria C. Kjellsson

**Affiliations:** 1 Department of Pharmacokinetics/Pharmacodynamics. Lilly Research Laboratories, Lilly Corporate Center, Indianapolis, IN 46285 USA; 2 Pharmacometrics Research Group, Department of Pharmacy, Uppsala University, Box 580, 751 23 Uppsala, Sweden

**Keywords:** Categorical modeling, Pharmacometrics, Preference, Hedonic, PKPD

## Abstract

The aim of this work was to develop and evaluate approaches of linked categorical models using individual predictions of probability. A model was developed using data from a study which assessed the perception of sweetness, creaminess, and pleasantness in dairy solutions containing variable concentrations of sugar and fat. Ordered categorical models were used to predict the individual sweetness and creaminess scores and these individual predictions were used as covariates in the model of pleasantness response. The model using individual predictions was compared to a previously developed model using the amount of fat and sugar as covariates driving pleasantness score. The model using the individual prediction of odds of sweetness and creaminess had a lower variability of pleasantness than the model using the content of sugar and fat in the test solutions, which indicates that the individual odds explain part of the variability in pleasantness. Additionally, simultaneous and sequential modeling approaches were compared for the linked categorical model. Parameter estimation was similar, but precision was better with sequential modeling approaches compared to the simultaneous modeling approach. The previous model characterizing the pleasantness response was improved by using individual predictions of sweetness and creaminess rather than the amount of fat and sugar in the solution. The application of this approach provides an advancement within categorical modeling showing how categorical models can be linked to enable the utilization of individual prediction. This approach is aligned with biology of taste sensory which is reflective of the individual perception of sweetness and creaminess, rather than the amount of fat and sugar in the solution.

**Supplementary Information:**

The online version contains supplementary material available at 10.1007/s10928-021-09771-y.

## Introduction

Overweight and obesity is a major, global health challenge [1]. Obesity is caused by a chronic positive energy balance; whereby adipose tissue is increased as a consequence of a positive balance between energy intake and utilization. Chronic over-eating, leading to obesity, is commonly categorized as an addictive behavior, similar to drug abuse, where craving and reward play an important role [2]. Meta-analysis of function MRI studies confirm this claim, showing that food and drugs activate similar brain regions [3]. Additionally, in both obesity and substance addition, similar brain abnormalities have been observed during the presentation of stimuli in reward and salience progressing [4]. One hypothesis is that the sensory perception of food in the mouth is closely linked to hedonic reward of feed and thus related to the motivation to eat [5]. Hedonic response is commonly measured using a sugar/fat preference test (SFPT) consisting of dairy solutions with varying content of fat and sugar [6–8]. Each dairy solution is rated for sweetness, creaminess, and pleasantness on separate visual analog scales.

Lewis Sheiner introduced, in 1994, the concept of nonlinear mixed-effects categorical data analysis in the field of pharmacokinetic-pharmacodynamic (PKPD) modeling [9]. It has since been used to characterize and quantify both clinical efficacy and side effects in a wide variety of therapeutics [10–13], including craving scores for smoking cessation [11]. Ordered categorical modeling is performed, assessing the probability of an observation, rather than the numerical value of the outcome itself, and the cumulative distribution of the outcome probability is commonly assumed to be a logistic distribution. The proportional odds model assumes that the covariate effect is the same across all categories, implying that a change in a covariate has the same effect on the log odds of all outcome categories [10]. The differential odds model relaxes this assumption and allows categories to be affected unequally by changes in a covariate [14]. The models are hierarchical as the differential odds model can collapse into the proportional odds model. The differential odds model has been shown to improve analysis of sedation data [14–16] and ocular itching scores [17].

Even though mixed-effects categorical models have been used for almost 25 years in the field of PKPD, linked categorical models, have to our knowledge, not been investigated. The principle of linking categorical models can be compared to a PKPD analysis, whereas the individual PK data informs the PD analysis through a model. Recently, traditional PKPD models have been expanded to PK-PD-PD models to include the relationship of a PD response driving another PD response, which is a common biology of hormonal regulation [18, 19]. The aim of this study was to develop linked categorical models, using the individual predictions of sweetness and creaminess from two separate categorical models as inputs into a differential odds model for the pleasantness response, and to compare the performance of this linked categorical model to a previously developed model which used the amount of sugar and fat in the solutions [20]. This was accomplished by using the same scoring data and assessing possible improvements between the models. Additionally, simultaneous and sequential modeling approaches were compared for the linked categorical model.

## Methods

### Clinical data

Data for modeling came from a single center study, approved by the Indiana University Institutional Review Board in 2006, and described in a previous paper by us [20]. All patients gave written informed consent and all procedures were performed in accordance with the ethical standards of the institutional research committee and Helsinki declaration.

Sixty-four healthy individuals (48% males) were categorized into three populations based on their body mass index (BMI): lean (BMI < 25), obese (30 ≤ BMI < 35), and very obese (BMI ≥ 35). No individuals were categorized as overweight (25 ≤ BMI < 30). All participating individuals were healthy, age 26–45 years old, with no known cardio-metabolic disease, drug dependency, essential medication or supplements. Individuals were also weight-stable, defined as no weight loss > 5 kg in last 6 months.

A SFPT was performed consisting of 16 dairy solutions containing: 0%, 3.5%, 11.3 or 37.5% fat by weight, with each fat level containing 0 %, 5%, 10%, or 20% sugar by weight. The study participants immediately rated each solution’s sweetness, creaminess, and pleasantness. All scales were anchored with descriptors with a score of 1 representing “not at all” and a score of 9 representing “extremely”. Two sessions of scorings of all 16 solutions were performed and the solutions were presented in random order within each scoring session. The method for rating the solutions was previously described [20].

### Model development

The scores of the SFPT were analyzed using mixed-effects modeling in NONMEM (version VII) (ICON Development Solutions) [21] and Perl-speaks-NONMEM (PsN) [22] as the modeling environment. In NONMEM, the Laplacian estimation method was used with the likelihood option. Model discrimination was based on visual predictive checks (VPCs), likelihood ratio test, based on objective function value (OFV; p-value = 0.01), and precision of parameter estimates. For two competing models with similar fit to the data (as judged by OFV), the simpler (i.e. fewer parameters) was selected. The VPCs were performed using PsN and Xpose in R [23], with 1000 study replicates (i.e., simulations) using the realized design of the original study, comparing incidence rates of sweetness, creaminess, and pleasantness scores.

### Sweetness and creaminess: individual predictions

The previously described models were used to predict the individual responses for sweetness and creaminess using proportional odds models [20]. The details of the models are given in supplementary material S1.

For sweetness, the covariates were both the amount of sugar and fat in the solution and the relationship was as follows:

$${g}_{S}\left(Sugar,Fat\right)=\frac{Sugar\cdot {S}_{max-Sugar}}{Sugar+{SSugar}_{50}}+{SL}_{S-Fat}\cdot Fat$$

where S_max−Sugar_ is the maximum increase in sweetness score with amount of sugar, SSugar_50_ is the amount of sugar giving half of S_max−Sugar_, and SL_S−Fat_ is the slope of the effect of amount of fat on the sweetness score. The relationship was additive to the logarithm of the odds of the scores in a proportional odds model, with an individual baseline sweetness for each, and inter-individual variability on S_max−Sugar_.

For creaminess, the covariates were the amount of sugar and fat in the solution, however the relationship was linear, as follows:

$${g}_{C}\left(Sugar,Fat\right)={SL}_{C-Fat}\cdot Fat+{SL}_{C-Sugar}\cdot Sugar$$

where SL_C−Fat_ and SL_C−Sugar_ are the slopes of the effect of the amount of fat and sugar, respectively, on the creaminess score. The relationship was additive to the logarithm of the odds of the scores in a proportional odds model, with individual baseline creaminess for each score.

### Pleasantness model

Pleasantness has previously been described by a differential odds model which allowed for sugar to be less than proportional and amount of fat to be greater than proportional on the pleasantness score [20]. The effect of the amount of sugar and fat on pleasantness was described by Emax models with a negative apparent interaction between sugar and fat [20]. This model was used for comparison to the model developed replacing sugar and fat content with the individual prediction of sweetness and creaminess. The model using content of sugar and fat is described by the following equations:

$${f}_{2}\left(Sugar,Fat\right)={\alpha }_{2}+{g}_{2}\left(Sugar,Fat\right)+{\eta }_{i}$$

$${f}_{3}\left(Sugar,Fat\right)={\alpha }_{2}+{\alpha }_{2\to 3}+{g}_{3}\left(Sugar,Fat\right)+{\eta }_{i}$$

$${f}_{4}\left(Sugar,Fat\right)={\alpha }_{2}+{\alpha }_{2\to 3}+{\alpha }_{3\to 4}+{g}_{4}\left(Sugar,Fat\right)+{\eta }_{i} (4)$$

…

$${f}_{9}\left(Sugar,Fat\right)={\alpha }_{2}+{\alpha }_{2\to 3}+{\alpha }_{3\to 4}+\dots +{\alpha }_{8\to 9}+{g}_{9}\left(Sugar,Fat\right)+{\eta }_{i}$$

where α_2_ describes log-odds corresponding to the probability of score ≥ 2, while α_x→x + 1_ with x = 2, 3, 4, 5, 6, 7, and 8 is the difference in log-odds between probability of score ≥ x and probability of score ≥ x + 1 and the functions g() were as follows:

$${g}_{2}\left(Sugar,Fat\right)=f\left(Sugar\right)+f\left(Fat\right)+Int\left(Sugar,Fat\right)$$

$${g}_{3}\left(Sugar,Fat\right)=f\left(Sugar\right)+f\left(Fat\right)\cdot {\beta }_{Fat3}+Int\left(Sugar,Fat\right)$$

$${g}_{4}\left(Sugar,Fat\right)=f\left(Sugar\right)+f\left(Fat\right)\cdot {\beta }_{Fat3}\cdot {\beta }_{Fat4}+Int\left(Sugar,Fat\right)$$

$${g}_{5/6}\left(Sugar,Fat\right)=f\left(Sugar\right)\cdot {\beta }_{Sugar5}+f\left(Fat\right)\cdot {\beta }_{Fat3}\cdot {\beta }_{Fat4}+Int\left(Sugar,Fat\right)$$

$${g}_{7/8/9}\left(Sugar,Fat\right)=f\left(Sugar\right)\cdot {\beta }_{Sugar5}+f\left(Fat\right)\cdot {\beta }_{Fat3}\cdot {\beta }_{Fat4}\cdot {\beta }_{Fat7}+Int\left(Sugar,Fat\right)$$

where the functions of sugar, fat and the apparent interactions were described by:

$$f\left(Sugar\right)=\frac{Sugar\cdot {P}_{max-Sugar}}{Sugar+{PSugar}_{50}}$$

$$f\left(Fat\right)=\frac{Fat\cdot {P}_{max-Fat}}{Fat+{PFat}_{50}}$$

where P_max−Sugar_ is the maximum increase in pleasantness score with amount of sugar, PSugar_50_ is the amount of sugar giving half of P_max−Sugar_, P_max−Fat_ is the maximum increase in pleasantness score with amount of fat, and PFat_50_ is the amount of fat giving half of P_max−Fat_.

$$Int\left(Sugar,Fat\right)={IP}_{Sugar-Fat}\cdot \left({wIP}_{Sugar}\frac{Sugar}{Max\left(Sugar\right)}+\frac{Fat}{Max\left(Fat\right)}\right)$$

For the apparent interaction between sugar and fat, Int(Sugar,Fat), the amount of sugar/fat was rescaled using the maximum investigated amounts, i.e. 37.5 % and 20 % for fat and sugar, respectively.

### Linked categorical modeling approaches

As with PKPD (concentration and effect) analysis, PK data can be fitted jointly to the PD response within a model. Within this study, two categorical models (sweetness and creaminess) were linked to a categorical model for pleasantness. The modeling approaches used to link the models are the same approaches used within PKPD analysis. These methods included a simultaneous modeling approach and two different sequential modeling approaches. To avoid repeating theory or method definitions in detail, we refer the reader to Zhang et al. [24]. The modeling approaches studied can be characterized qualitatively as follows:

#### Simultaneous approach (SIM)

During the simultaneous approach, the parameters for all models (creaminess, sweetness and pleasantness) were estimated at the same time, as the maximum of the joint likelihood for all data. This approach allows for the information within all models to influence each other, and accounts for the uncertainty across the models.

#### Sequential approaches

In sequential approaches, the parameters for creaminess and sweetness were estimated separately from the pleasantness. These parameters were then used as fixed parameters in the model for pleasantness, estimating pleasantness parameters using the individual predictions for creaminess and sweetness. The way the individual predictions are generated is different based on the approach used.

- In the Population (PK) Parameter and Data (PPP&D) approach, the individual predictions for sweetness and creaminess originated from the fixed population parameters and the assigned individual deviations from the central trend. These deviations were informed by the sweetness and creaminess data and assigned as the pleasantness parameters were estimated.
- In the Individual (PK) Parameter (IPP) approach, the individual predictions of sweetness and creaminess were generated prior to the estimation of pleasantness parameters and added as covariates for the individuals in the dataset used for estimation of pleasantness parameters.

## Results

### Pleasantness model using individual prediction of sweetness and creaminess

Various structural models for pleasantness were explored (such as linear, inhibitor Emax and sigmoidal Emax models), as well as different transformations of the individual predictions. Use of the individual predictions of sweetness and creaminess were explored in the model as they were (range −∞ to ∞), exponentiated (range 0 to ∞) or logit-transformed (range 0–1). The same structural model used previously for the content of fat and sugar was found to best fit the data, replacing the content of sugar and fat with the individual odds of sweetness and creaminess, respectively, anchored on score 1:

$$f\left(Sweet\right)=\frac{{\text{O}\text{d}\text{d}\text{s}}_{{Sweet}_{i}}\cdot {P}_{max-Sweet}}{{\text{O}\text{d}\text{d}\text{s}}_{{Sweet}_{i}}+{PSweet}_{50}}$$

$$f\left(Cream\right)=\frac{{\text{O}\text{d}\text{d}\text{s}}_{{Cream}_{i}}\cdot {P}_{max-Cream}}{{\text{O}\text{d}\text{d}\text{s}}_{{Cream}_{i}}+{PCream}_{50}}$$

$$Int\left(Sweet,Cream\right)={IP}_{Sweet\text{-}Cream}\cdot \left({wIP}_{Sugar}\frac{{\text{O}\text{d}\text{d}\text{s}}_{{Sweet}_{i}}}{\text{10,000}}+\frac{{\text{O}\text{d}\text{d}\text{s}}_{{Cream}_{i}}}{\text{10,000}}\right)$$

The individual odds of sweetness and creaminess, anchored on score 1, were derived as the exponent of the individual predictions of the log-odds of the cumulative probability of sweetness/creaminess scores > 1. The individual odds were rescaled, similar to the amounts, when used in the function for apparent interactions between the covariates. However, where the amounts were rescaled with the maximum amounts, the exponents of individual predictions were rescaled arbitrarily with 10,000. As two sessions of scorings were performed, inter-occasion variability (IOV) was explored within the model.

### Comparison between linked model with sweetness and creaminess and the model with amounts of sugar and fat

**Fig. 1:**
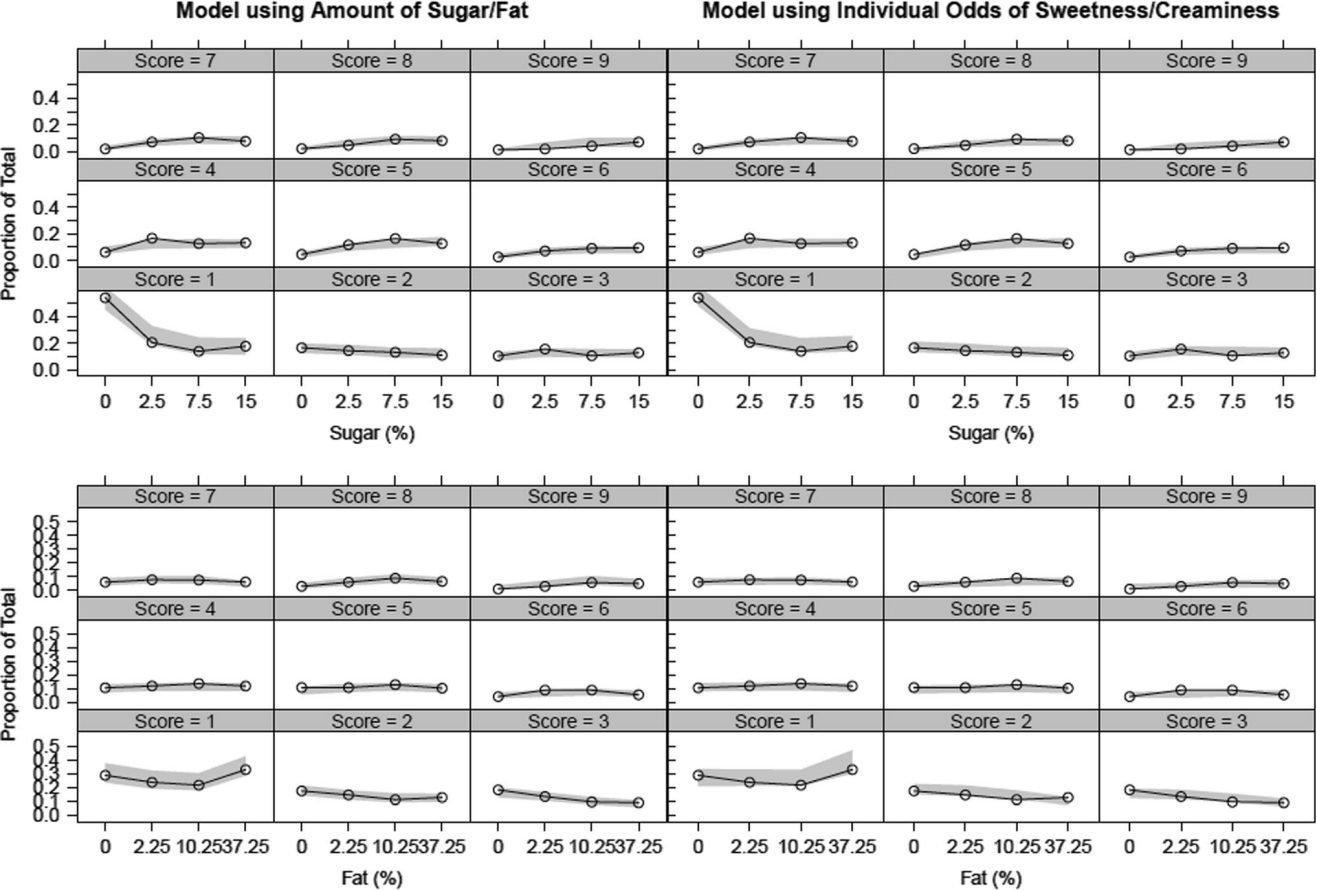
Visual predictive checks for the pleasantness scale based on a nine-category scales for each of solution of the SFPT. Lines represent the proportions (nine‐category scale) binned by either the amount of sugar (top panels) or amount of fat (bottom panels) and the areas are the corresponding 95 % confidence intervals from 1000 simulations using the model’s final parameter estimates for the two models (left panels: model using amount of sugar and fat; right panel: Linked model using individual prediction of sweetness and creaminess)

Based on the Akaike Information Criterion (AIC), the model with the lower OFV reflects a better fit of the data when the number of parameters between compared models are the same. As the two models used the same scoring data, number of model parameters and the relationship between the probability and the predictor was conserved between models. The difference in OFV and precision in parameter estimates were used to compare the model using individual predictions of sweetness and creaminess to the model using amounts of sugar and fat. Importantly, only the input for the predictor (sugar vs. sweetness or fat vs. creaminess) differed between these models. Using a simultaneous approach, a model improvement (ΔOFV − 9) was observed with the model using individual prediction of sweetness and creaminess compared to the model using the content of sugar and fat. Correspondingly, there was slightly greater certainty in most parameter estimates using individual prediction of sweetness and creaminess (Table 1). Additionally, the variance of pleasantness was reduced from 4.21 using the model the content of sugar and fat to 3.2 using the model individual prediction of sweetness and creaminess. The VPCs for the pleasantness score shows a similar fit of the data for model using individual prediction of sweetness and creaminess compared to the model using the content of sugar and fat (Fig. 1 and Figure S1). This is unsurprising given that VPC for categorical data is rather insensitive in visualizing small differences in predictive properties between models. The VPCs for the sweetness and creaminess score from model using individual prediction of sweetness and creaminess are also provided in the Supplemental (Fig. S2). The addition of IOV was also explored within the model and only marginally improved (ΔOFV − 6) the fit of the model using the amount of sugar and fat or the linked model using individual predictions of sweetness and creaminess.

**Table 1:** List of parameter estimates with uncertainty (relative standard error—RSE) for the model using amount of fat and sugar (*f(Amount)*) and individual predictions of sweetness and creaminess (*f(Odds*_*pred,i*_*)*) to assess pleasantness

	Parameter description	Parameter	*f(Amount)*		*f(Odds*_*pred,i*_ *)*	
			Estimate	RSE (%)	Estimate	RSE (%)
Sweetness score	Logit of score >1	*α*_*S1*_	− 0.764	70	− 0.599	61
	Logit of score =2	*α*_*S2->3*_	− 1.12	10	− 1.11	11
	Logit of score =3	*α*_*S3->4*_	− 1.00	13	− 0.977	11
	Logit of score =4	*α*_*S4->5*_	− 0.829	9.1	− 0.806	11
	Logit of score =5	*α*_*S5->6*_	− 0.740	9.9	− 0.722	8.6
	Logit of score =6	*α*_*S6->7*_	− 0.797	10.7	− 0.78	8.9
	Logit of score =7	*α*_*S7->8*_	− 0.979	8.0	− 0.963	7.4
	Logit of score =8	*α*_*S8->9*_	− 1.23	6.6	− 1.21	6.0
	Maximal effect of sugar	*S*_*max-Sugar*_	8.32	4.9	8.23	4.3
	Sugar giving half of S_max,Sugar_	*SSugar*_*50*_	7.89	14	8.42	12
	Slope of fat effect	*SL*_*S-Fat*_	0.004	140	0.00332	103
Creaminess score	Logit of score >1	*α*_*C1*_	1.06	62	1.15	53
	Logit of score =2	*α*_*C2->3*_	− 1.46	6.0	− 1.43	6.1
	Logit of score =3	*α*_*C3->4*_	− 1.06	8.9	− 1.05	10.2
	Logit of score =4	*α*_*C4->5*_	− 0.83	11	− 0.817	11.4
	Logit of score =5	*α*_*C5->6*_	− 0.846	13	− 0.834	13.3
	Logit of score =6	*α*_*C6->7*_	− 0.902	14	− 0.893	12.7
	Logit of score =7	*α*_*C7->8*_	− 1.12	15	− 1.12	14.9
	Logit of score =8	*α*_*C8->9*_	− 1.43	11	− 1.45	10.9
	Slope of fat effect	*SL*_*C-Fat*_	0.186	14	0.183	14.4
	Slope of sugar effect	*SL*_*C-Sugar*_	0.049	16	0.0379	25.1
Pleasantness score	Logit of score >1	*α*_*P1*_	− 0.482	165	− 1.38	52
	Logit of score =2	*α*_*P2->3*_	− 1.36	11	− 1.96	20
	Logit of score =3	*α*_*P3->4*_	− 1.2	14	− 1.5	16
	Logit of score =4	*α*_*P4->5*_	− 0.865	6.6	− 0.904	8.8
	Logit of score =5	*α*_*P5->6*_	− 0.627	35	− 0.407	90
	Logit of score =6	*α*_*P6->7*_	− 0.661	13	− 0.656	14
	Logit of score =7	*α*_*P7->8*_	− 1.21	19	− 1.63	37
	Logit of score =8	*α*_*P8->9*_	− 1.34	12	− 1.32	12
	Maximal effect of sugar/sweet	*P*_*max-Sugar/Sweet*_	7.96	105	4.21	5.5
	Sugar/sweet giving half of P_max-Sugar/Sweet_	*PSugar*_*50*_ */ PSweet*_*50*_	9.86	120	5.62	43
	Maximal effect of fat/cream	*P*_*max-Fat/Cream*_	2.41	61	0.466	60
	Fat/cream giving half P_max-Fat/Cream_	*PFat*_*50*_ */ PCream*_*50*_	8.71	72	1.65	70
	Interaction of sugar/sweet and fat/cream	*IP*_*Sugar-Fat*_ */ IP*_*Sweet-Cream*_	− 2.31	44	− 1.91	94
	Weight of sugar/sweet on interaction	*wIP*_*Sugar/Sweet*_	1.06	160	39.5	109
	Differential effect ≥5, sugar	*β*_*Sugar5*_	0.928	7.7	0.864	10
	Differential effect ≥3, fat	*β*_*Fat3*_	1.23	12	3.3	62
	Differential effect ≥4, fat	*β*_*Fat4*_	1.26	14	1.5	17
	Differential effect ≥7, fat	*β*_*Fat7*_	1.19	11	1.4	20
Variance	Sweetness baseline*	*w* ^*2*^_*S*_	1.34	35	1.3	36
	Creaminess baseline*	*w* ^*2*^_*C*_	4.21	31	4.03	38
	Pleasantness baseline*	*w* ^*2*^_*P*_	4.21	28	3.2	28
	S_max-Sugar_	*w* ^*2*^_*Smax-Sugar*_	0.207	33	0.207	33

### Comparison of approaches for the linked model with sweetness and creaminess

**Fig. 2:**
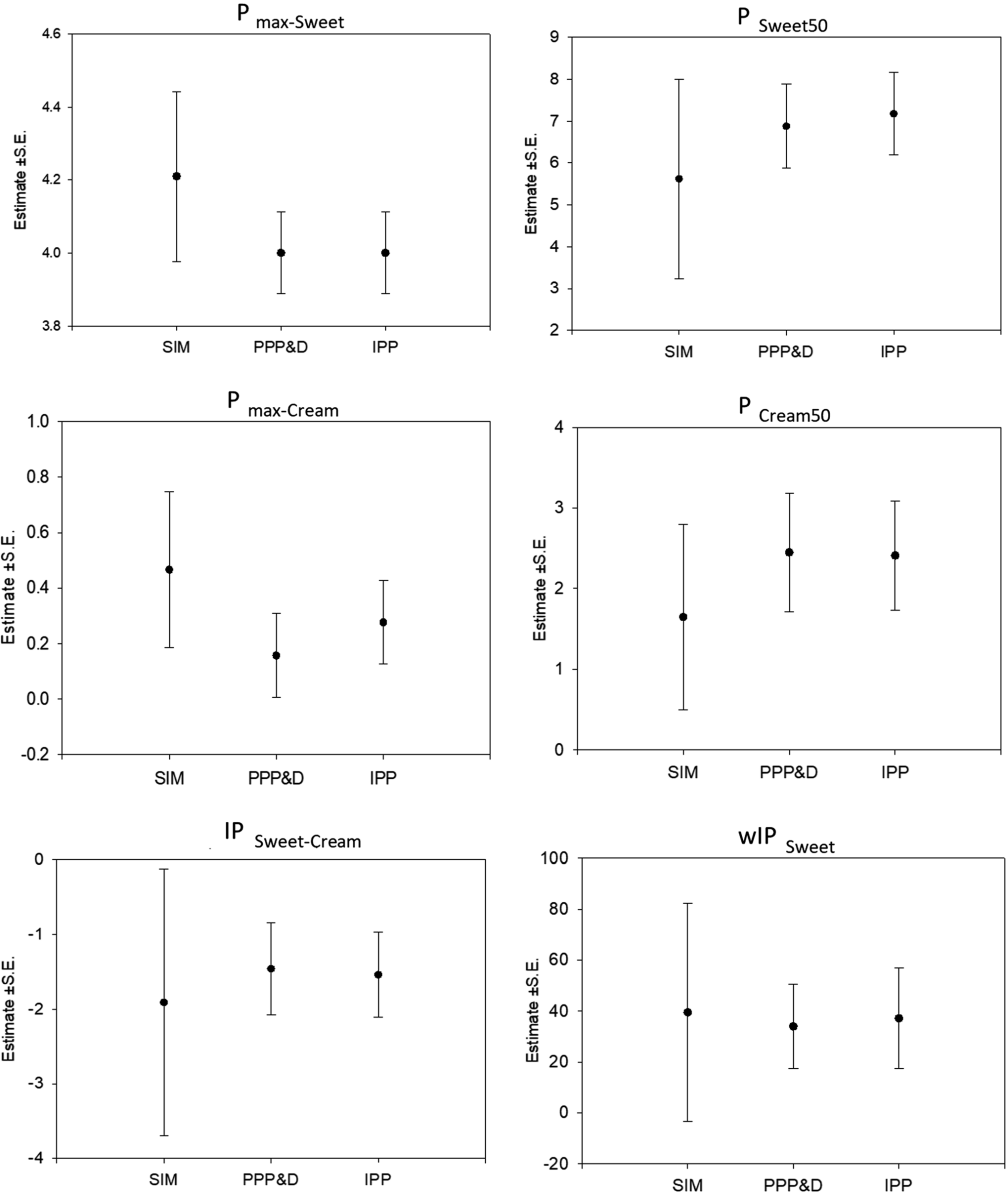
Pleasantness parameter estimates and standard errors for the different linked modeling approaches: simultaneous (SIM), fixed population parameters and data (PPP&D), and individual predicted parameters (IPP)

For the linked model using individual predictions of sweetness and creaminess to assess pleasantness, the three approaches, IPP, PPP&D and SIM were compared in terms of the estimates and uncertainties of the pleasantness parameters, P_max−sweet_, PSweet_50_, P_max−cream_, PCream_50_, IP_sweet−cream_, and wIP_sweet_ (Fig. 2).

The standard errors overlapped for the different pleasantness parameter estimates across the different modeling approaches. The model estimate precision, as measured by the standard error, was consistently smaller for the sequential methods (PPP&D and IPP) compared to SIM. The PPP&D and IPP approaches had similar parameter estimates and standard errors.

## Discussion

This was the first study to develop a linked categorical model using the individual predictions from proportional odds models of creaminess and sweetness as input into a differential odds model for pleasantness response. This analysis shows that it is possible to link categorical models, as commonly performed for PKPD analysis. This approach is particularly attractive for taste perception studies, as the rating of the pleasantness would be more related to the individual’s perception of the sweetness and creaminess of the solution, rather than the amount of fat and sugar. Correspondingly, there was an improvement in the model when using individual predictions compared to the amount of sugar and fat in the test solutions during a simultaneous modeling approach. Similar to PKPD modeling, we found that using the individual prediction of sweetness and creaminess reduced the estimate of variability of pleasantness compared to the model using the amount of sugar and fat to describe the pleasantness score.

Replacing the content of sugar and fat in the solutions with the individual scores of sweetness and creaminess in the model showed the best fit (ΔOFV − 409). This proves that the perception of sweetness and creaminess are better predictors of pleasantness than the amount of sugar and fat in the solution, and this model can be viewed as the best-case scenario. Notably, the variability of pleasantness with the model using the scores of sweetness and creaminess was the same as with the linked model (i.e., the model using individual predictions of odds). Thus, it appears as if the fit with the individual predictions of odds is sufficient to account for the variability, although it does not represent an equally good fit as the observed scores of sweetness and creaminess (ΔOFV + 400). Worth mentioning, the model using the observed scores does not provide an understanding of the implication of how the amount of sugar and fat influences the perception of pleasantness and thus, is less useful than the linked model in that context.

The predictions of sweetness and creaminess could be linked to the pleasantness in different ways. In addition to the presented linked model using the individual predicted odds anchored on score 1, a linked model using the individual prediction of the score was investigated. The individual prediction of score was derived as the probability of a score multiplied with the size of the score, i.e.,

$${I}_{pred}={Prob}_{1}\cdot 1+{Prob}_{2}\cdot 2+\dots +{Prob}_{9}\cdot 9$$

Although, this prediction of the sweetness/creaminess represented a prediction of the same scale as the observed scores, this model did not improve the fit (ΔOFV + 1.8). Thus, it appears as if the linked model is rather insensitive to how the link was implemented. With PKPD modeling, there are various approaches to jointly analyze the population PK and PD observations. Both SIM and sequential approaches (PPP&D and IPP) [24, 25] were assessed in linking the categorical models within this study. The SIM, PPP&D and IPP approaches provided similar estimates for the pleasantness parameters. The model estimate precision was consistently smaller for the sequential methods (PPP&D and IPP) compared to SIM. This is consistent with evaluation of PK/PD modeling approaches by Zhang et al. [23] which found that standard error estimates were larger with SIM than the corresponding values estimated with the sequential methods. Overall, the three methods produced similar outcomes, which may not be surprising given the large amount of data within this study supporting the analysis. These methods would be more likely to differ with sparse data which may allow for model misspecifications [24, 25]. Lastly, a simultaneous fit may not gain additional advantage over our sequential methods within this study, as the measures of sweetness and creaminess are uncorrelated. Likewise, the relationship is one-directional where the perception of creaminess and sweetness would likely inform the pleasantness response, rather than perception of pleasantness informing the perception of creaminess and sweetness.

The limitations within this analysis are relative to the methodology. This study indicates that these linked modeling approaches are comparable; however, other datasets with different sizes and complexities need to be assessed to further explore this analysis. Additionally, other biological linked categorical relationships should be assessed.

## Conclusions

The pleasantness score was improved by using individual predictions of sweetness and creaminess within the model rather than the amount of fat and sugar in the solution. Application of this modeling approach provides an advancement within categorical modeling whereas categorical models can be linked to enable the utilization of individual prediction. This approach is more aligned to the biology of taste sensory which is reflective of the individual perception of the sweetness and creaminess, rather than the amount of fat and sugar in the solution.

## Supplementary Information

Below is the link to the electronic supplementary material.

Supplementary file1 (DOCX 127 kb)
