# Supplementary material for: Linking categorical models for prediction of pleasantness score using individual predictions of sweetness and creaminess: An advancement of categorical modeling
Source: J Pharmacokinet Pharmacodyn. 2021 Jul 1;48(6):815–23. doi: 10.1007/s10928-021-09771-y (PMC8604822; doi:10.1007/s10928-021-09771-y)
Supplement: Supplementary file 1 — Supplementary file1 (DOCX 127 kb) [file 10928_2021_9771_MOESM1_ESM.docx]

Supplementary material S1 – Proportional Odds Model

The proportional odds model is described below. If *y_i_* = *(*y*_i_* _1_*,* y*_i_* _2_*, . . .,*y*_i 32_)* is the vector of categorical response for the *i*^th^ individual with *32* observations, then the probability that an observation, *y_in_* is greater than or equal to the score *j* (where *j= 2 … 9*) has the following general structure:

$$Pr\left( y_{in}\geq j | \eta_{i} \right)=\frac{e^{{logit}_{ij}}}{1+e^{{logit}_{ij}}}$$

where

$${logit}_{ij}=f_{j}\left( \right);j=2,\ldots$$

The function, *f_j_( )* describes baseline probabilities (on logit scale) and effects of predictors, e.g. covariates, doses and time, as follows:

$$f_{2}\left( \right)=\alpha_{2}+g\left( X \right)+ \eta_{i}$$

$$f_{3}\left( \right)=\alpha_{2}+\alpha_{2\to3}+g\left( X \right)+ \eta_{i}$$

$$f_{4}\left( \right)=\alpha_{2}+\alpha_{2\to3}+\alpha_{3\to4}+g\left( X \right)+ \eta_{i}$$

*…*

$$f_{9}\left( \right)=\alpha_{2}+\alpha_{2\to3}+\alpha_{3\to4}+\ldots+\alpha_{8\to9}+g\left( X \right)+ \eta_{i}$$

where*η_i_* is the random effect, describing inter-individual differences. All random effects belong to a normal distribution with zero mean and variance *ω*^2^, which is estimated.  *X* represents predictors and α are baseline probabilities. In this particular project, *X* was the amount of sugar and fat in the test solutions for the comparison model of pleasantness, while *X* was the exponent of the individual prediction of sweetness and creaminess for the linked categorical pleasantness model.

The probabilities of the individual scores are then derived as follows:

$$Pr\left( y_{in}=1 | \eta_{i} \right)=1-Pr\left( y_{in}\geq2 | \eta_{i} \right)$$

$$Pr\left( y_{in}=2 | \eta_{i} \right)=Pr\left( y_{in}\geq2 | \eta_{i} \right)-Pr\left( y_{in}\geq3 | \eta_{i} \right)$$

$$Pr\left( y_{in}=3 | \eta_{i} \right)=Pr\left( y_{in}\geq3 | \eta_{i} \right)-Pr\left( y_{in}\geq4 | \eta_{i} \right)$$

$$Pr\left( y_{in}=4 | \eta_{i} \right)=Pr\left( y_{in}\geq4 | \eta_{i} \right)-Pr\left( y_{in}\geq5 | \eta_{i} \right)$$

$$Pr\left( y_{in}=5 | \eta_{i} \right)=Pr\left( y_{in}\geq5 | \eta_{i} \right)-Pr\left( y_{in}\geq6 | \eta_{i} \right)$$

$$Pr\left( y_{in}=6 | \eta_{i} \right)=Pr\left( y_{in}\geq6 | \eta_{i} \right)-Pr\left( y_{in}\geq7 | \eta_{i} \right)$$

$$Pr\left( y_{in}=7 | \eta_{i} \right)=Pr\left( y_{in}\geq7 | \eta_{i} \right)-Pr\left( y_{in}\geq8 | \eta_{i} \right)$$

$$Pr\left( y_{in}=8 | \eta_{i} \right)=Pr\left( y_{in}\geq8 | \eta_{i} \right)-Pr\left( y_{in}\geq9 | \eta_{i} \right)$$

$Pr\left( y_{in}=9 | \eta_{i} \right)=Pr\left( y_{in}\geq9 | \eta_{i} \right)$

Figure S1 Visual predictive check for pleasantness. Solid, black lines represent the median pleasantness score and dotted, gray lines represent the 2.5th and 97.5th percentiles of score by either the amount of sugar (top panels) or amount of fat (bottom panels) and the areas are the corresponding 95% confidence intervals from 1,000 simulations using the model's final parameter estimates for the two models (left panels: model using amount of sugar and fat; right panel: Linked model using individual prediction of sweetness and creaminess).

Figure S2 Visual predictive checks for creaminess (right) and sweetness (left) and based on a nine‐category scales for each of solution of the SFPT. Lines represent the proportions (nine‐category scale) binned by either the amount of fat or sugar, and the areas are the corresponding 95% confidence intervals from 1,000 simulations from the linked model using individual prediction of sweetness and creaminess.

Supplement: Model File

$INPUT ID SWT CRM DV CMT

$DATA data.csv

$PRED

;Parameter definition

BS1 = THETA(1); baseline

BS2 = THETA(2) ; reduced sweetness logit of score = 2

BS3 = THETA(3) ; reduced sweetness logit of score = 3

BS4 = THETA(4) ; reduced sweetness logit of score = 4

BS5 = THETA(5) ; reduced sweetness logit of score = 5

BS6 = THETA(6) ; reduced sweetness logit of score = 6

BS7 = THETA(7) ; reduced sweetness logit of score = 7

BS8 = THETA(8) ; reduced sweetness logit of score = 8

BC1 = THETA(9) ; baseline creaminess logit of score >1 (i.e 2,3,4,5,6,7,8,9)

BC2 = THETA(10) ; reduced creaminess logit of score = 2

BC3 = THETA(11) ; reduced creaminess logit of score = 3

BC4 = THETA(12) ; reduced creaminess logit of score = 4

BC5 = THETA(13) ; reduced creaminess logit of score = 5

BC6 = THETA(14) ; reduced creaminess logit of score = 6

BC7 = THETA(15) ; reduced creaminess logit of score = 7

BC8 = THETA(16) ; reduced creaminess logit of score = 8

BP1 = THETA(17) ; baseline pleasantness logit of score >1 (i.e 2,3,4,5,6,7,8,9)

BP2 = THETA(18) ; reduced pleasantness logit of score = 2

BP3 = THETA(19) ; reduced pleasantness logit of score = 3

BP4 = THETA(20) ; reduced pleasantness logit of score = 4

BP5 = THETA(21) ; reduced pleasantness logit of score = 5

BP6 = THETA(22) ; reduced pleasantness logit of score = 6

BP7 = THETA(23) ; reduced pleasantness logit of score = 7

BP8 = THETA(24) ; reduced pleasantness logit of score = 8

EMAX = THETA(25) ; EMAX sugar on sweetness

EC50 = THETA(26) ; EC50 sugar on sweetness

EFC = THETA(27) ; fat on creaminess

ESPMAX = THETA(28) ;EMAX sweetness on pleasantness

ESPEC = THETA(29) ;EC50 sweetness on pleasantness

EFPMAX = THETA(30) ;EMAX creaminess on pleasantness

EFPEC = THETA(31) ;EC50 creaminess on pleasantness

EIP = THETA(32) ;interaction term for sweetness and creaminess

EIPSC = THETA(33) ;scaler for interaction for sweetness and creaminess

HILL= THETA(34); Hill coefficient for sweetness on pleasantness

betaS1 = THETA(35) ;EMAX Scaler1 sweetness on pleasantness

betaS2 = THETA(36) ;EMAX Scaler2 sweetness on pleasantness

betaS3 = THETA(37) ;EMAX Scaler3 sweetness on pleasantness

betaS4 = THETA(38) ;EMAX Scaler4 sweetness on pleasantness

betaS5 = THETA(39) ;EMAX Scaler5 sweetness on pleasantness

betaS6 = THETA(40) ;EMAX Scaler6 sweetness on pleasantness

betaS7 = THETA(41) ;EMAX Scaler7 sweetness on pleasantness

betaC1 = THETA(42) ;EMAX Scaler1 creaminess on pleasantness

betaC2 = THETA(43) ;EMAX Scaler2 creaminess on pleasantness

betaC3 = THETA(44) ;EMAX Scaler3 creaminess on pleasantness

betaC4 = THETA(45) ;EMAX Scaler4 creaminess on pleasantness

betaC5 = THETA(46) ;EMAX Scaler5 creaminess on pleasantness

betaC6= THETA(47) ;EMAX Scaler6 creaminess on pleasantness

betaC7 = THETA(48) ;EMAX Scaler7 creaminess on pleasantness

ESS = THETA(50) ; fat on sweetness

EFS = THETA(49) ; sugar on creaminess

;Sweetness

IPREDS=ETA(1)+ CRM*ESS + ((SWT*EMAX)/(SWT+EC50))

LGT1S = BS1+IPREDS ; logit for cumulative scores for sweetness>1

LGT2S = LGT1S+BS2 ; logit for cumulative scores for sweetness>2

LGT3S = LGT2S+BS3 ; logit for cumulative scores for sweetness>3

LGT4S = LGT3S+BS4 ; logit for cumulative scores for sweetness>4

LGT5S = LGT4S+BS5 ; logit for cumulative scores for sweetness 5

LGT6S = LGT5S+BS6 ; logit for cumulative scores for sweetness>6

LGT7S = LGT6S+BS7 ; logit for cumulative scores for sweetness>7

LGT8S = LGT7S+BS8 ; logit for cumulative scores for sweetness >8 => score=9

EIPREDS=EXP(IPREDS)

PGT1S = EXP(LGT1S)/(1+EXP(LGT1S)) ; Prob of cumulative score >1

PGT2S = EXP(LGT2S)/(1+EXP(LGT2S)) ; Prob of cumulative score >2

PGT3S = EXP(LGT3S)/(1+EXP(LGT3S)) ; Prob of cumulative score >3

PGT4S = EXP(LGT4S)/(1+EXP(LGT4S)) ; Prob of cumulative score >4

PGT5S = EXP(LGT5S)/(1+EXP(LGT5S)) ; Prob of cumulative score >5

PGT6S = EXP(LGT6S)/(1+EXP(LGT6S)) ; Prob of cumulative score >6

PGT7S = EXP(LGT7S)/(1+EXP(LGT7S)) ; Prob of cumulative score >7

PGT8S = EXP(LGT8S)/(1+EXP(LGT8S)) ; Prob of cumulative score >8

P1S = 1-PGT1S ;Probability of 1

P2S = PGT1S-PGT2S ;Probability of 2

P3S = PGT2S-PGT3S ;Probability of 3

P4S = PGT3S-PGT4S ;Probability of 4

P5S = PGT4S-PGT5S ;Probability of 5

P6S = PGT5S-PGT6S ;Probability of 6

P7S = PGT6S-PGT7S ;Probability of 7

P8S = PGT7S-PGT8S ;Probability of 8

P9S = PGT8S ;Probability of 9

;Creaminess

IPREDC=ETA(2) + SWT*EFS + CRM*EFC *EXP(ETA(4))

LGT1C = BC1+IPREDC ; logit for cumulative scores for creaminess>1

LGT2C = LGT1C+BC2 ; logit for cumulative scores for creaminess >2

LGT3C = LGT2C+BC3 ; logit for cumulative scores for creaminess >3

LGT4C = LGT3C+BC4 ; logit for cumulative scores for creaminess >4

LGT5C = LGT4C+BC5 ; logit for cumulative scores for creaminess >5

LGT6C = LGT5C+BC6 ; logit for cumulative scores for creaminess >6

LGT7C = LGT6C+BC7 ; logit for cumulative scores for creaminess >7

LGT8C = LGT7C+BC8 ; logit for cumulative scores for creaminess >8 => score=9

EIPREDC=EXP(IPREDC)

PGT1C = EXP(LGT1C)/(1+EXP(LGT1C)) ; Prob of cumulative score >1

PGT2C = EXP(LGT2C)/(1+EXP(LGT2C)) ; Prob of cumulative score >2

PGT3C = EXP(LGT3C)/(1+EXP(LGT3C)) ; Prob of cumulative score >3

PGT4C = EXP(LGT4C)/(1+EXP(LGT4C)) ; Prob of cumulative score >4

PGT5C = EXP(LGT5C)/(1+EXP(LGT5C)) ; Prob of cumulative score >5

PGT6C = EXP(LGT6C)/(1+EXP(LGT6C)) ; Prob of cumulative score >6

PGT7C = EXP(LGT7C)/(1+EXP(LGT7C)) ; Prob of cumulative score >7

PGT8C = EXP(LGT8C)/(1+EXP(LGT8C)) ; Prob of cumulative score >8

P1C = 1-PGT1C ;Probability of 1

P2C = PGT1C-PGT2C ;Probability of 2

P3C = PGT2C-PGT3C ;Probability of 3

P4C = PGT3C-PGT4C ;Probability of 4

P5C = PGT4C-PGT5C ;Probability of 5

P6C = PGT5C-PGT6C ;Probability of 6

P7C = PGT6C-PGT7C ;Probability of 7

P8C = PGT7C-PGT8C ;Probability of 8

P9C = PGT8C ;Probability of 9

;Pleasantness

LGT1P = BP1 + ETA(3) + EIP*(EIPSC*(EIPREDS/1E5)+(EIPREDC/1E5)) + ((ESPMAX*EIPREDS**HILL)/(ESPEC**HILL+EIPREDS**HILL)) + ((EIPREDC*EFPMAX)/(EIPREDC+EFPEC))

LGT2P = BP1 + BP2 + ETA(3) + EIP*(EIPSC*(EIPREDS/1E5)+(EIPREDC/1E5)) + ((ESPMAX*betaS1*EIPREDS**HILL)/(ESPEC**HILL+EIPREDS**HILL)) + ((EIPREDC*EFPMAX*betaC1)/(EIPREDC+EFPEC))

LGT3P = BP1 + BP2 + BP3 + ETA(3) + EIP*(EIPSC*(EIPREDS/1E5)+(EIPREDC/1E5)) + ((ESPMAX*betaS1*betaS2*EIPREDS**HILL)/(ESPEC**HILL+EIPREDS**HILL))+((EIPREDC*EFPMAX*betaC1*betaC2)/(EIPREDC+EFPEC))

LGT4P = BP1 + BP2 + BP3 + BP4 + ETA(3) + EIP*(EIPSC*(EIPREDS/1E5)+(EIPREDC/1E5)) + ((ESPMAX*betaS1*betaS2*betaS3*EIPREDS**HILL)/(ESPEC**HILL+EIPREDS**HILL)) + ((EIPREDC*EFPMAX*betaC1*betaC2*betaC3)/(EIPREDC+EFPEC))

LGT5P = BP1 + BP2 + BP3 + BP4 + BP5 + ETA(3) + EIP*(EIPSC*(EIPREDS/1E5)+(EIPREDC/1E5)) + ((ESPMAX*betaS1*betaS2*betaS3*betaS4*EIPREDS**HILL)/(ESPEC**HILL+EIPREDS**HILL)) + ((EIPREDC*EFPMAX*betaC1*betaC2*betaC3*betaC4)/(EIPREDC+EFPEC))

LGT6P = BP1 + BP2 + BP3 + BP4 + BP5 + BP6 + ETA(3)+EIP*(EIPSC*(EIPREDS/1E5)+(EIPREDC/1E5))+ ((ESPMAX*betaS1*betaS2*betaS3*betaS4*betaS5*EIPREDS**HILL)/(ESPEC**HILL+EIPREDS**HILL))+((EIPREDC*EFPMAX*betaC1*betaC2*betaC3*betaC4*betaC5)/(EIPREDC+EFPEC))

LGT7P = BP1 + BP2 + BP3 + BP4 + BP5 + BP6 + BP7 + ETA(3) + EIP*(EIPSC*(EIPREDS/1E5)+(EIPREDC/1E5)) + ((ESPMAX*betaS1*betaS2*betaS3*betaS4*betaS5*betaS6*EIPREDS**HILL)/(ESPEC**HILL+EIPREDS**HILL))+((EIPREDC*EFPMAX*betaC1*betaC2*betaC3*betaC4*betaC5*betaC6)/(EIPREDC+EFPEC))

LGT8P = BP1 + BP2 + BP3 + BP4 + BP5 + BP6 + BP7 + BP8 + ETA(3) + EIP*(EIPSC*(EIPREDS/1E5)+(EIPREDC/1E5)) + ((ESPMAX*betaS1*betaS2*betaS3*betaS4*betaS5*betaS6*betaS7*EIPREDS**HILL)/(ESPEC**HILL+EIPREDS**HILL)) + ((EIPREDC*EFPMAX*betaC1*betaC2*betaC3*betaC4*betaC5*betaC6*betaC7)/(EIPREDC+EFPEC))

PGT1P = EXP(LGT1P)/(1+EXP(LGT1P)) ; Prob of cumulative score >1

PGT2P = EXP(LGT2P)/(1+EXP(LGT2P)) ; Prob of cumulative score >2

PGT3P = EXP(LGT3P)/(1+EXP(LGT3P)) ; Prob of cumulative score >3

PGT4P = EXP(LGT4P)/(1+EXP(LGT4P)) ; Prob of cumulative score >4

PGT5P = EXP(LGT5P)/(1+EXP(LGT5P)) ; Prob of cumulative score >5

PGT6P = EXP(LGT6P)/(1+EXP(LGT6P)) ; Prob of cumulative score >6

PGT7P = EXP(LGT7P)/(1+EXP(LGT7P)) ; Prob of cumulative score >7

PGT8P = EXP(LGT8P)/(1+EXP(LGT8P)) ; Prob of cumulative score >8

P1P = 1-PGT1P ;Probability of 1

P2P = PGT1P-PGT2P ;Probability of 2

P3P = PGT2P-PGT3P ;Probability of 3

P4P = PGT3P-PGT4P ;Probability of 4

P5P = PGT4P-PGT5P ;Probability of 5

P6P = PGT5P-PGT6P ;Probability of 6

P7P = PGT6P-PGT7P ;Probability of 7

P8P = PGT7P-PGT8P ;Probability of 8

P9P = PGT8P ;Probability of 9

IF(DV.EQ.1.AND.CMT.EQ.2) Y = P1S ;Probability of 1 on sweetness

IF(DV.EQ.2.AND.CMT.EQ.2) Y = P2S ;Probability of 2 on sweetness

IF(DV.EQ.3.AND.CMT.EQ.2) Y = P3S ;Probability of 3 on sweetness

IF(DV.EQ.4.AND.CMT.EQ.2) Y = P4S ;Probability of 4 on sweetness

IF(DV.EQ.5.AND.CMT.EQ.2) Y = P5S ;Probability of 5 on sweetness

IF(DV.EQ.6.AND.CMT.EQ.2) Y = P6S ;Probability of 6 on sweetness

IF(DV.EQ.7.AND.CMT.EQ.2) Y = P7S ;Probability of 7 on sweetness

IF(DV.EQ.8.AND.CMT.EQ.2) Y = P8S ;Probability of 8 on sweetness

IF(DV.EQ.9.AND.CMT.EQ.2) Y = P9S;Probability of 9 on sweetness

IF(DV.EQ.1.AND.CMT.EQ.1) Y = P1C ;Probability of 1 on creaminess

IF(DV.EQ.2.AND.CMT.EQ.1) Y = P2C ;Probability of 2 on creaminess

IF(DV.EQ.3.AND.CMT.EQ.1) Y = P3C ;Probability of 3 on creaminess

IF(DV.EQ.4.AND.CMT.EQ.1) Y = P4C ;Probability of 4 on creaminess

IF(DV.EQ.5.AND.CMT.EQ.1) Y = P5C ;Probability of 5 on creaminess

IF(DV.EQ.6.AND.CMT.EQ.1) Y = P6C ;Probability of 6 on creaminess

IF(DV.EQ.7.AND.CMT.EQ.1) Y = P7C ;Probability of 7 on creaminess

IF(DV.EQ.8.AND.CMT.EQ.1) Y = P8C ;Probability of 8 on creaminess

IF(DV.EQ.9.AND.CMT.EQ.1) Y = P9C ;Probability of 9 on creaminess

IF(DV.EQ.1.AND.CMT.EQ.3) Y = P1P ;Probability of 1 on pleasantness

IF(DV.EQ.2.AND.CMT.EQ.3) Y = P2P ;Probability of 2 on pleasantness

IF(DV.EQ.3.AND.CMT.EQ.3) Y = P3P ;Probability of 3 on pleasantness

IF(DV.EQ.4.AND.CMT.EQ.3) Y = P4P ;Probability of 4 on pleasantness

IF(DV.EQ.5.AND.CMT.EQ.3) Y = P5P ;Probability of 5 on pleasantness

IF(DV.EQ.6.AND.CMT.EQ.3) Y = P6P ;Probability of 6 on pleasantness

IF(DV.EQ.7.AND.CMT.EQ.3) Y = P7P ;Probability of 7 on pleasantness

IF(DV.EQ.8.AND.CMT.EQ.3) Y = P8P ;Probability of 8 on pleasantness

IF(DV.EQ.9.AND.CMT.EQ.3) Y = P9P ;Probability of 9 on pleasantness

$THETA (-3,-0.718749) ; BS1

$THETA (-3,-1.11277,0) ; BS2

$THETA (-3,-1.02871,0) ; BS3

$THETA (-3,-0.834546,0) ; BS4

$THETA (-3,-0.7272,0) ; BS5

$THETA (-3,-0.782712,0) ; BS6

$THETA (-3,-0.97185,0) ; BS7

$THETA (-3,-1.19676,0) ; BS8

$THETA (0,1.03588) ; BC1

$THETA (-3,-1.46303,0) ; BC2

$THETA (-3,-1.058,0) ; BC3

$THETA (-3,-0.829936,0) ; BC4

$THETA (-3,-0.842895,0) ; BC5

$THETA (-3,-0.899397,0) ; BC6

$THETA (-3,-1.13231,0) ; BC7

$THETA (-3,-1.4536,0) ; BC8

$THETA -1.15772 ; BP1

$THETA (-3,-1.86027,0) ; BP2

$THETA (-3,-1.50335,0) ; BP3

$THETA (-3,-0.896659,0) ; BP4

$THETA (-3,-0.457289,0) ; BP5

$THETA (-3,-0.659535,0) ; BP6

$THETA (-3,-1.64615,0) ; BP7

$THETA (-3,-1.29546,0) ; BP8

$THETA 8.32149 ; EMAX 25

$THETA 7.89433 ; EC50 26

$THETA 0.186391 ; EFC 27

$THETA (0,4.21246) ; ESPMAX 28

$THETA (0,6.14804) ; ESPEC 29

$THETA (0,0.0699856) ; EFPMAX 30

$THETA (0,2.25125) ; EFPEC 31

$THETA -1.01395 ; EIP 32

$THETA (0,61.833) ; EIPSC 33

$THETA 1 FIX ; HILL 34

$THETA 1 FIX ; betaS1

$THETA 1 FIX ; betaS2

$THETA 1 FIX ; betaS3

$THETA (0,0.877851,1) ; betaS4

$THETA 1 FIX ; betaS5

$THETA 1 FIX ; betaS6

$THETA 1 FIX ; betaS7

$THETA (0,14.9252,15) ; betaC1

$THETA (0,1.75821,2) ; betaC2

$THETA 1 FIX ; betaC3

$THETA 1 FIX ; betaC4

$THETA 1 FIX ; betaC5

$THETA (0,1.51539,1.6) ; betaC6

$THETA 1 FIX ; betaC7

$THETA 0.0491906 ; slope of sugar on creaminess

$THETA 0.00361282 ; slope of fat on sweetness

$OMEGA 1.3448 ; SWT

$OMEGA 4.21106 ; Fat

$OMEGA 3.31404 ; PL

$OMEGA 0.206624 ; EFC_

$ESTIMATION MAXEVAL=9999 METH=COND LAPLACE LIKE

$COVARIANCE PRINT=E MATRIX=S

Example Input file:

| CID | SWT | CRM | DV | CMT |
| --- | --- | --- | --- | --- |
| 7001 | 0 | 0 | 1 | 1 |
| 7001 | 0 | 0 | 1 | 2 |
| 7001 | 0 | 0 | 1 | 3 |
| 7001 | 5 | 0 | 3 | 1 |
| 7001 | 5 | 0 | 3 | 2 |
| 7001 | 5 | 0 | 2 | 3 |
| 7001 | 10 | 0 | 3 | 1 |
| 7001 | 10 | 0 | 3 | 2 |
| 7001 | 10 | 0 | 3 | 3 |
| 7001 | 20 | 0 | 3 | 1 |
| 7001 | 20 | 0 | 4 | 2 |
| 7001 | 20 | 0 | 3 | 3 |
| 7001 | 0 | 3.5 | 1 | 1 |
| 7001 | 0 | 3.5 | 1 | 2 |
| 7001 | 0 | 3.5 | 1 | 3 |
| 7001 | 5 | 3.5 | 1 | 1 |
| 7001 | 5 | 3.5 | 2 | 2 |
| 7001 | 5 | 3.5 | 1 | 3 |
| 7001 | 10 | 3.5 | 2 | 1 |
| 7001 | 10 | 3.5 | 2 | 2 |
| 7001 | 10 | 3.5 | 2 | 3 |
| 7001 | 20 | 3.5 | 3 | 1 |
| 7001 | 20 | 3.5 | 4 | 2 |
| 7001 | 20 | 3.5 | 2 | 3 |
| 7001 | 0 | 11.3 | 1 | 1 |
| 7001 | 0 | 11.3 | 1 | 2 |
| 7001 | 0 | 11.3 | 1 | 3 |
| 7001 | 5 | 11.3 | 1 | 1 |
| 7001 | 5 | 11.3 | 1 | 2 |
| 7001 | 5 | 11.3 | 1 | 3 |
| 7001 | 10 | 11.3 | 2 | 1 |
| 7001 | 10 | 11.3 | 1 | 2 |
| 7001 | 10 | 11.3 | 2 | 3 |
| 7001 | 20 | 11.3 | 2 | 1 |
| 7001 | 20 | 11.3 | 4 | 2 |
| 7001 | 20 | 11.3 | 3 | 3 |
| 7001 | 0 | 37.5 | 1 | 1 |
| 7001 | 0 | 37.5 | 1 | 2 |
| 7001 | 0 | 37.5 | 1 | 3 |
| 7001 | 5 | 37.5 | 3 | 1 |
| 7001 | 5 | 37.5 | 3 | 2 |
| 7001 | 5 | 37.5 | 2 | 3 |
| 7001 | 10 | 37.5 | 3 | 1 |
| 7001 | 10 | 37.5 | 3 | 2 |
| 7001 | 10 | 37.5 | 2 | 3 |
| 7001 | 20 | 37.5 | 3 | 1 |
| 7001 | 20 | 37.5 | 4 | 2 |
| 7001 | 20 | 37.5 | 2 | 3 |
